# Novel rhesus macaque immunoglobulin germline genes identified by three sequencing approaches

**DOI:** 10.3389/fimmu.2024.1506348

**Published:** 2024-12-24

**Authors:** Yicheng Guo, Eric Waltari, Hong Lu, Zizhang Sheng, Xueling Wu

**Affiliations:** ^1^ Aaron Diamond AIDS Research Center, Columbia University Vagelos College of Physicians and Surgeons, New York, NY, United States; ^2^ Division of Infectious Diseases, Department of Medicine, Columbia University Vagelos College of Physicians and Surgeons, New York, NY, United States

**Keywords:** rhesus macaque, IGHV genes, NGS, database, antibody

## Abstract

**Introduction:**

Rhesus macaques have long been a focus of research for understanding immune responses to human pathogens due to their close phylogenetic relationship with humans. As rhesus macaque antibody germlines show high degrees of polymorphism, the spectrum of database-covered genes expressed in individual macaques remains to be determined.

**Methods:**

Here, four rhesus macaques infected with SHIV_SF162P3N_ became a study of interest because they developed broadly neutralizing antibodies against HIV-1. To identify the immunoglobulin heavy chain V-gene (IGHV) germlines in these macaques, we applied three sequencing approaches – genomic DNA (gDNA) TOPO sequencing, gDNA MiSeq, and messenger RNA (mRNA) MiSeq inference with IgDiscover, and illustrated the detection power of each method.

**Results:**

Of the 197 new rhesus IGHV germline sequences identified, 116 (59%) were validated by at least two methods, and 143 (73%) were found in at least two macaques or two sample sources. About 20% of germlines in each macaque are missing from the current database, including a subset frequently expressed. Overall, gDNA MiSeq determined the greatest number of germline sequences, followed by gDNA TOPO sequencing and mRNA MiSeq inference by IgDiscover, with IgDiscover providing direct evidence of allele expression and usage.

**Discussion:**

Our interdisciplinary study sheds light on germline sequencing, enhances the rhesus IGHV germline database, and highlights the importance of germline sequencing in rhesus immune repertoire studies.

## Introduction

Due to high levels of genetic similarity to humans, rhesus macaques (*Macaca mulatta*) have served as vital models for pre-clinical investigations and vaccine development for various human diseases, including infectious diseases (1–5), neurological disorders (6–8), and immunological conditions (9, 10). As a pivotal component of the adaptive immune response, B cells and B cell receptors (BCRs) recognize and bind to foreign antigens, leading to specific B cell activation and production of functional antibodies. Characterization of rhesus macaque antibodies for antigen binding specificity, affinity maturation, and protection mechanisms provides valuable insights to vaccine and therapeutics research against human pathogens (11–13).

The enormous diversity of BCRs – approximately 10^9^ clonal types – is crucial for recognizing a vast array of foreign antigens and mounting effective antibody responses (14, 15). BCRs use the variable domains of both heavy and light chains to recognize antigens, and the BCR variable domain is generated through V(D)J recombination, which is the primary determinant of BCR diversity. Unlike humans, rhesus macaque immunoglobulin (IG) germline genes are highly polymorphic, requiring the identification of IG germline genes from individual macaques (16–18). Alternatively, a comprehensive rhesus germline database could serve as a germline reference and expedite antibody studies in rhesus macaques (19–21). The most up-to-date database KIMDB (21) includes 774 IGHV alleles from 27 rhesus macaques, 15 with Indian and 12 with Chinese origins. However, it remains unclear whether additional germline genes and alleles are beneficial for the comprehensiveness of a rhesus IGHV database.

Advancements in sequencing and computational technologies have enabled a faster and more comprehensive collection of IG genes from various species, including mice, rats, rabbits, dogs, bovines, camels, macaques, gorillas, and humans (22). The conventional approach is to bulk PCR amplify IG genes from genomic DNA (gDNA) followed by TOPO cloning and Sanger sequencing (TOPO sequencing) (23–26). This approach is the most accurate but labor-intensive and low throughput, limited to hundreds of sequencing reads. Alternatively, the IG gene amplicons can be sequenced by next-generation sequencing (NGS) technology. However, NGS systematic errors raise uncertainties and undermine the confidence and accuracy of the IG genes assembled. Genomic sequencing of the IG locus can also obtain IG gene sequences, but the features of the IG locus such as large size, sequence diversity, duplication, deletion, and repetitive structure impede confident identification of all IG genes, especially those assembled from short-read NGS (27–29). Finally, IG gene prediction software (IgDiscover (30) and IMPre (31)) enables the identification of IG genes from expressed naïve BCR repertoires, typically obtained through messenger RNA (mRNA) NGS, with the drawback of missing IG genes infrequently expressed, and the VDJ gene rearrangement can make the ends of IGHV alleles harder to identify. Nonetheless, the mRNA-based inference approaches not only predict germline alleles but also provide the significant advantage of confirming allele expression and usage. Hence, a systematic comparison of these different approaches will provide insights into the optimal technologies that identify rhesus IG genes with high accuracy and good coverage.

Here, we applied three approaches to identify IGHV germline sequences from four rhesus macaques − two with Indian and two with Chinese origins, which were studied for neutralizing antibody responses to simian-human immunodeficiency virus (SHIV) infection (32, 33). We compared the results from gDNA-derived PCRs followed by TOPO sequencing or MiSeq, and those obtained from expressed IgM transcripts coupled with IgDiscover. Our data revealed IGHV genes or alleles identified by one or more of these methods. We also examined the IgDiscover-predicted germlines from an additional four rhesus macaques as a case study for germline coverages by current databases. In total, we present 444 rhesus IGHV germline sequences from four rhesus macaques, with 197 not found in KIMDB, some of which were frequently expressed in macaque BCR repertoires. This highlights the importance of determining individual macaque-specific germline genes to improve the coverage and accuracy of antibody ontogeny analysis. The new germlines provide an updated valuable resource for the scientific community. The availability of this dataset will undoubtedly advance B cell biology in non-human primates and pave the way for better-informed comparative studies with humans.

## Methods

### Rhesus macaque specimens

The four-rhesus peripheral blood mononuclear cell (PBMC) samples analyzed in this study were from SHIV_SF162P3N_-infected macaques (two Indian and two Chinese) described previously (32–38). The macaque samples were collected at the Tulane National Primate Research Center, in compliance with Guide for the Care and Use of Laboratory Animals and under protocols approved by the Institutional Animal Care and Use Committee (IACUC), with animal welfare assurance number A3081-01, approval date 8 October 2012.

### FACS, gDNA extraction, and gDNA PCR

Rhesus PBMCs were isolated using Ficoll-Pacque, with ACK buffer lysing red blood cells. FACS was performed to collect non-B cells. Briefly, about 5 million PBMCs were stained with antibodies to CD3-PE-CF594 (BD Biosciences, San Jose, CA) and CD20-APC-Cy7 (BioLegend). In addition, live/dead yellow stain (Invitrogen) was used to exclude dead cells. After washing, cells were sorted using a multi-laser MoFlo sorter (Beckman Coulter, Jersey City, NJ). Fluorescence compensation was performed with anti-mouse IG kappa chain beads (BD Biosciences) stained with each antibody in a separate tube. CD3^+^ non-B cells were bulk sorted for gDNA extraction. The cellular gDNA was extracted using the AllPrep DNA/RNA Mini Kit (Qiagen), with a final elution in 200 μL buffer. The V-genes of the IG heavy chain locus were amplified by multiple PCRs, each targeting one to two IGHV gene families, either from the 5’ untranslated region (5UTR) or the leader region, using mixed Pfu and Taq polymerases.

Primer sequences:

1  rh_IGHV3_leader  5’ GGG GCT GAG CTG GGT TTT CCT TGT TGC 3’

2  rh_IGHV3_leaderA  5’ GGG GCT GAG CTG GAT TTT CCT TGT TGC 3’

3  rh_IGHV3_leaderT 5’ GGG GCT GAG CTT GGT TTT CCT TGT TGC 3’

4  rh_IGHV3_5UTR  5’ GAC YCT GCA GCT CTG GGA GAG G 3’

5  rh_IGHV3_5UTR_A1  5’ GAC YCT GCA GCT CTG GGA GAA G 3’

6  rh_IGHV3_5UTR_A2  5’ GAC YCT GCA GCT CTG AGA GAG G 3’

7  rh_IGHV3_5UTR_AC  5’ GAC YCT GCA GCT CTG AGA CAG G 3’

8  rh_IGHV3_5UTR_CA  5’ GAC YCT GCA GCT CCG AGA GAG G 3’

9  rh_IGHV3_3UTR  5’ TTT GTG TCT GGG CTC ACA CTG ACC TC 3’

10  rh_IGHV3_3UTR_A1  5’ TTT GTG TCT GGG CTC ACA CTG ACT TC 3’

11  rh_IGHV3_3UTR_A2  5’ TTT GTG TCT GGG CTC ACA TTG ACC TC 3’

12  rh_IGHV1_leader  5’ GAT CCT CCT CTT GGT GGC AGC A 3’

13  rh_IGHV1_leaderA  5’ GAT CCT CCT CTT GGT GAC AGC A 3’

14  rh_IGHV7_leader  5’ TTC TTG GTG GCA GCA GCA ACA 3’

15  rh_IGHV1_5UTR  5’ GAG AGC ATC ACA CAA CAA CCA CA 3’

16  rh_IGHV1_5UTR2  5’ GAG AAC ATC ACT CAA CAA CTG CA 3’

17  rh_IGHV7_5UTR  5’ GCC CTG AGA GCA TCA CCC AAC A 3’

18  rh_IGHV1_3UTR  5’ TTC TGA CAC TCT CAG GAT GTG G 3’ (IGHV7 included)

19  rh_IGHV4_leader  5’ CTG TGG TTC TTC CTC CTC CTG 3’

20  rh_IGHV4_leader_T  5’ CTG TGG TTC TTC TTC CTC CTG 3’

21  rh_IGHV6_leader  5’ GCA ATG TCT GTC TCC TTC CTC ATC 3’

22  rh_IGHV4_5UTR  5’ TCA CAT GGG AAA TGY TCT CTG AGA 3’

23  rh_IGHV6_5UTR  5’ CCT GCT GAA TTC CGG CTA ACC A 3’

24  rh_IGHV4_3UTR  5’ GGA GGT TTG TGT CTG GGC TCA 3’ (IGHV6 included)

25  rh_IGHV2_leader  5’ CTT TGC TCC ACG CTC CTG CT 3’

26  rh_IGHV2_5UTR  5’ ATC TCC ACC AGC TCC ACC CT 3’

27  rh_IGHV2_3UTR  5’ CAG CCT GGG TTC TTG TAC AGG A 3’

28  rh_IGHV5_leader  5’ TTC TGT CCT CCA CCA CGA TGG G 3’

29  rh_IGHV5_leader_TG 5’ TTC TGT CCT CCT CCA GGA TGG G 3’

30  rh_IGHV5_5UTR  5’ ACC GCA CAG CTG GGA TCT CA 3’

31  rh_IGHV5_3UTR  5’ AGG GAG TCT CTG GCA GCT CA 3’

32  rh_IGHV5_3UTR2  5’ AGG GTG TCT CTG GCA CCT CA 3’

PCR cycling conditions were 98°C for 1 min, 25 cycles of 94°C for 15 sec, 52°C for 30 sec, and 72°C for 1 min, followed by 72°C for 10 min. PCR amplicons were inspected on 2% agarose gel revealing sizes from 450 to 550 base pairs, then extracted and purified using the QIAquick Gel Extraction Kit (Qiagen).

### TOPO cloning and Sanger sequencing

PCR products were cloned into the vector Blunt II-TOPO (Invitrogen Life Technologies). Colonies were miniprepped (Qiagen), and then subjected to Sanger sequencing by Macrogen (Rockville, MD). Sequences were examined using Sequencher v5.3 and Geneious v8.1. All chromatograms were inspected for quality and mixed bases (double peaks), which would be evidence of priming from more than one template or PCR error in early cycles. Any sequence with evidence of double peaks was excluded from further analysis. We examined sequences for stop codons and examined the 5’ and 3’ UTR surrounding the V-gene of each sequence to confirm presence of both leader and V-gene recombination signals.

### Deep sequencing of gDNA and sequence analysis

The same sets of IGHV PCRs amplified from gDNA were pooled for Illumina MiSeq library preparation and 2 x 300 bp paired-end indexed sequencing at the New York Genome Center, with PCR samples from all 4 animals multiplexed in a single run. The raw 2 x 300 paired end reads from Illumina MiSeq were assembled into single reads using USEARCH (39). We then used USEARCH to calculate the theoretical total numbers of miscalls based on Q scores for each sequence and excluded reads with more than 10 potential miscalls. SONAR (40) was then used to annotate the IGHV genes. Briefly, reads shorter than 300 nucleotides were removed. Customized BLAST (https://www.ncbi.nlm.nih.gov/blast+) was used to assign germline IGHV to each read with customized parameters (40) with KIMDB as reference database for each animal. The open-reading frame was then determined and the sequence regions other than IGHV were removed, including the nucleotides at the 3’ end after the conserved “CAR” or similar amino acid motif. Sequencing reads containing frameshift and/or stop codons likely due to low quality or sequencing errors were excluded. To further remove PCR duplicates and sequencing errors, we clustered full-length reads with 100% nucleotide identity, using USEARCH with a similar strategy used in our previous studies (41, 42). Here, we chose USEARCH because it offers search and clustering algorithms that are often orders of magnitude faster than BLAST.

### Deep sequencing of mRNA and IgDiscover

As previously described (43, 44), about 5 million rhesus PBMC mRNA was extracted using the Oligotex Direct mRNA Mini Kit (Qiagen), eluted in 200 μL buffer and concentrated to 10-25 μL using Ultra 0.5 mL Centrifugal filters (Amicon). For 5’ RACE cDNA synthesis, each 10 μL mRNA was mixed with 1 μL Oligo dT_12-18_ at 12 μM (Life Technologies) at 70°C for 1 min and then -20°C for 1 min, followed by addition of 1 μL SMARTer Oligo at 12 μM (Clontech), 4 μL 5× first-strand buffer, 1 μL DTT at 20 mM, 1 μL dNTP at 10 mM each, 1 μL RNaseOUT, and 1-3 μL SuperScript II (Life Technologies). The mixtures were incubated at 42°C for 2 hours and then passed through a PCR cleanup spin column (Machery-Nagel).

From a single cDNA template equivalent to transcripts of 4-5 million PBMCs, the variable regions of μ chain alone (IgM-only), or with γ and α heavy chain, or adding κ and λ light chains, were amplified using the KAPA HiFi qPCR kit (KAPA Biosystems) with a universal 5’ primer IIA (Clontech).

Primer sequences:

1  5’ IIA  5’ AAG CAG TGG TAT CAA CGC AGA G 3’

2  3’ Mu-R  5’ ATT CTC ACA GGA GAC GAG GGG GAA AAG GGT TG 3’

3  3’ Gamma-R  5’ GGG GAA GAC CGA TGG GCC CTT GGT GGA RG 3’

4  3’ Alpha-R_rh  5’ CGG GAA GAC CTT GGG TTT GGT CGG 3’

5  3’ Kappa-R_rh  5’ GGA AGA TGA AGA CAG ATG GTG SAG CCA CAG 3’

6  3’ Lambda-R_rh  5’ CCY TGT TGG CTT GAA GCT CCT CAG AGG AGG 3’

PCR cycling conditions were 98°C for 45 sec, 18-25 cycles of 98°C for 15 sec, 65°C for 30 sec, and 72°C for 45 sec, followed by 72°C for 3 min. PCR products were loaded on 2% E-gels (Life Technologies) for visualization and extraction at the size around 450 base pairs, with a final buffer exchange using the PCR Micro Kit (Life Technologies). The eluted PCR was used for Illumina MiSeq library preparation and 2 x 300 bp paired-end indexed sequencing at the New York Genome Center, with two PCR samples multiplexed per run.

Raw 2 x 300 paired-end reads from Illumina MiSeq were then used as input for SONAR to assemble full length V(D)J transcripts (40). The output nucleotide sequences were then used as input for IgDiscover (30) version 0.15.1 with default parameters. Germline genes from KIMDB (21) and the Ramesh et al. study (45) was employed as reference databases. New alleles identified from iteration one of each IgDiscover run were used in this study. The sequence regions other than IGHV were removed, including the nucleotides at the 3’ end after the conserved “CAR” or similar amino acid motif.

### Sequence data deposit

Each new gene or allele was named with the gene template followed by ‘W’ plus four digits – W_series. The W_series, IMGT (22), and KIMDB were combined to form an updated gene database KIMDB_W with a total of 1,111 germline alleles. ‘Union’ and ‘Rename’ functions in IgDiscover package were utilized to build the combined KIMDB_W database and then rename all sequences. The final N=1,111 KIMDB_W containing the N=197 W_series new rhesus macaque IGHV germline sequences (**Supplementary Table S1**) is available in GenBank under accession numbers PQ429293 to PQ430403, as well as at the figshare link:

https://figshare.com/articles/dataset/Combined_Rhesus_Macaque_germline_database/27170985?file=49615155. The gDNA and mRNA MiSeq data have been deposited to the NCBI SRA database under accession number PRJNA1181892.

## Results

### Cohort and experimental design

**Figure 1:**
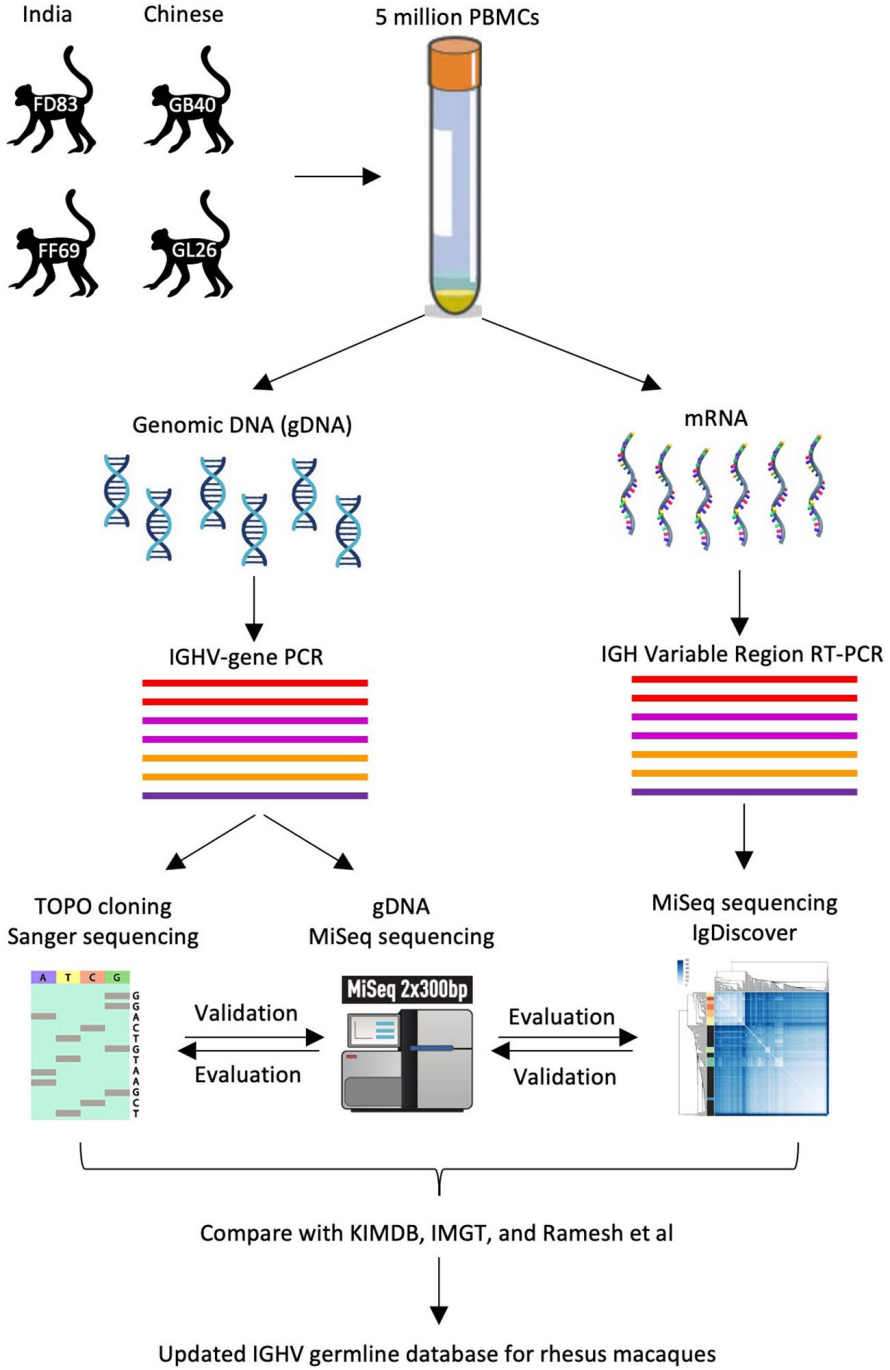
Workflow of three IGHV sequencing approaches. As illustrated, three sequencing approaches – namely, gDNA TOPO sequencing, gDNA MiSeq, and mRNA MiSeq with IgDiscover, are applied to samples from four rhesus macaques to obtain their IGHV germline sequences.

A cohort of four rhesus macaques – FD83 and FF69 with Indian origin, GB40 and GL26 with Chinese origin – were infected with SHIV_SF162P3N_ and mounted broadly neutralizing antibody responses (32, 33). To facilitate the antibody analyses of these animals, we aimed to determine their IGHV germline genes (**Figure 1**). About five million PBMCs (or more than one million blood CD3^+^ non-B cells) from these animals were lysed for gDNA and mRNA extraction. The gDNA was used to amplify the IGHV genes by multiple PCRs, each targeting one or two IGHV gene families, from the 5’ UTR or the leader region. The gDNA-derived PCR amplicons were then subjected to TOPO sequencing and MiSeq for the identification of IGHV germline genes. The mRNA was used for RT-PCR to amplify the variable region of expressed BCR transcripts. The naïve BCR transcripts were then subjected to MiSeq and IgDiscover for the identification of IGHV germline genes. The germline results obtained by the three methods – TOPO sequencing, MiSeq, and IgDiscover – were compared and used to update the existing databases.

### Germline IGHV sequences identified by gDNA TOPO sequencing

The gDNA-derived PCRs spanning individual V-genes of the IG heavy chain locus were TOPO cloned for Sanger sequencing to capture the full length of IGHV genes. A total of N = 249 to 280 high-quality sequences were obtained from each animal (**Table 1**). After eliminating redundant and non-productive reads, N = 81 to 90 unique IGHV sequences were determined from each macaque. Pairwise comparison of these TOPO sequences revealed that only one allele (IGHV6-1*02) was shared among these four macaques (**Supplementary Figure S1A**). Additionally, 43 alleles were shared by at least two macaques, while 230 alleles belonged to specific individual macaques. Furthermore, there was no preferential overlap of germline alleles between Chinese and Indian macaques.

**Table 1:** Results from gDNA TOPO sequencing.

Macaque	Origin	SHIV infection (weeks)	Sample type	Colonies sequenced	TOPO_unique sequences
FD83	Indian	5	CD3^+^ non-B	267	84
FF69	Indian	42	PBMC	278	84
GB40	Chinese	13	PBMC	249	81
GL26	Chinese	4, 5, 23	CD3^+^ non-B	280	90

### Germline IGHV sequences identified by gDNA MiSeq

**Figure 2:**
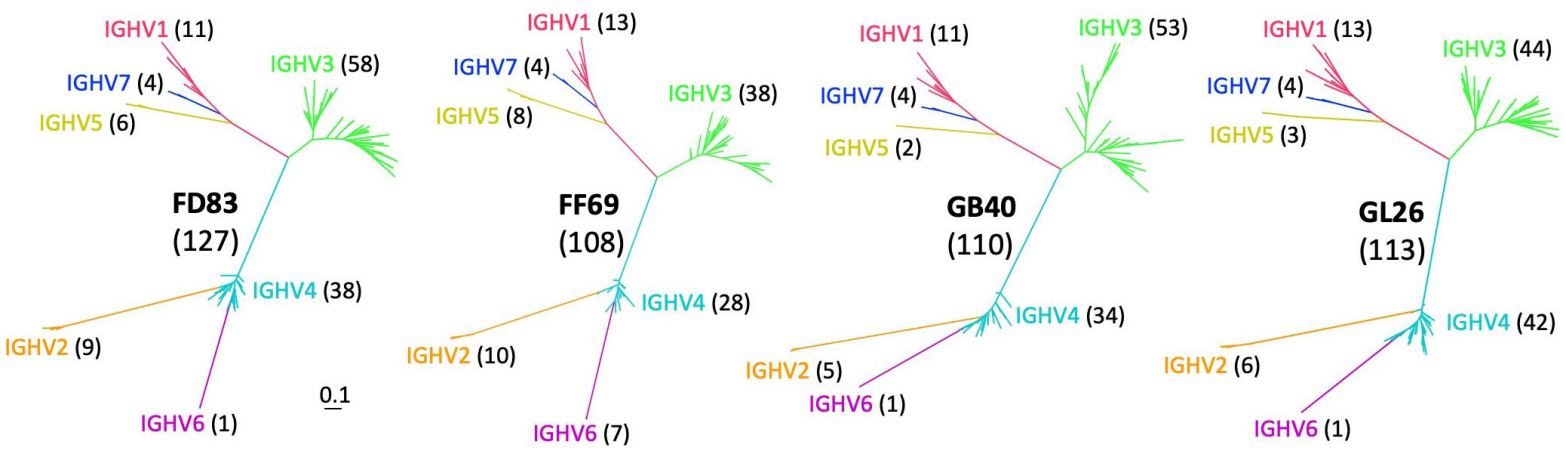
Phylogenetic trees of germline alleles identified from gDNA MiSeq in each macaque. The gene families are highlighted in red, orange, green, cyan, yellow, purple, and blue, corresponding to IGHV1 through IGHV7, respectively. The number in parentheses indicates the total number of alleles in each animal and gene family. .

The same sets of gDNA-derived PCRs spanning individual V segments of the IG heavy chain locus were subjected to MiSeq sequencing, yielding N = 4.7 to 13.3 million raw reads from each animal (**Table 2**). Over 11,000 unique IGHV sequences were acquired from each macaque, with the highest sequencing depth reaching over 49,000 copies. To account for potential sequencing errors, we took the unique reads and ranked them by sequencing depth, which dropped around the ~120th sequence in each monkey (**Supplementary Figure S1B**) to depths approximately 0.67 to 1.0% of the maximum. Consequently, we set a threshold at 0.67% (> 1,000 reads) of maximum sequencing depth to identify confident IGHV alleles for each macaque. This threshold resulted in N = 140, 114, 116, and 126 IGHV sequences for FD83, FF69, GB40, and GL26, respectively. We acknowledge that this threshold may be affected by sequencing depth and PCR efficiency of the MiSeq library thus may not be optimal to all studies. After excluding non-productive sequences, the final IGHV germline sequences were N = 127, 108, 110, and 113 for the respective macaques (**Table 2**). We utilized these germline sequences to construct a maximum likelihood tree for each macaque using the general time reversible (GTR) model. The trees were annotated by comparing the IGHV sequences to the established germline genes from IMGT/GENE-DB (https://www.imgt.org/). The phylogenetic trees displayed a consistent topological structure across all macaques, with IGHV3 being the most predominant family, followed by IGHV4, IGHV1, and IGHV2 (**Figure 2**). Notably, fewer than 10 alleles were found in the IGHV5, IGHV6, and IGHV7 families in each macaque, aligning with previous studies (19, 46).

**Table 2:** Results from gDNA MiSeq.

Macaque	Origin	SHIV infection (weeks)	Raw reads	Assembled	Merged	Confident	Unique	Maximum depth	Final
FD83	Indian	5	4,746,677	3,119,736	3,117,903	3,027,232	11,195	49,574	127
FF69	Indian	42	13,299,409	9,640,951	9,636,783	9,352,891	32,470	285,429	108
GB40	Chinese	13	12,124,347	9,033,988	9,030,584	8,527,186	25,842	831,331	110
GL26	Chinese	4, 5, 23	5,822,435	3,752,378	3,751,252	3,187,554	12,387	91,745	113

### Germline IGHV sequences predicted by IgM transcripts and IgDiscover

IgDiscover (30) is a software that predicts IGHV germline genes from the IgM transcript sequencing data. IgDiscover aligns sequences to known IGHV germlines and examines within the sequences assigned to an allele for novel alleles. It evaluates the number of J alleles and CDR3s associated with a candidate IGHV allele to discriminate true germline polymorphisms from somatic hypermutations (SHM) and sequencing errors. Here, beyond gDNA libraries, we also obtained the mRNA MiSeq data of the heavy chain variable domain from the four macaques studied. Using the IgM-only MiSeq reads, we employed IgDiscover to predict the IGHV germline sequences for each macaque. We used the heavy chain genes in KIMDB (21) as reference, and the algorithm predicted N = 83 to 107 alleles from three rhesus macaques (**Table 3**). For FF69, IgDiscover was applied to the expressed heavy chain transcripts comprising of IgM, IgG, and IgA; with a minimal number of IgM reads in an isotype mixed repertoire, IgDiscover predicted N = 31 IGHV germline alleles in FF69 (**Table 3**). Further comparison showed that 76 (83%), 25 (81%), 84 (79%), and 73 (88%) of IgDiscover predicted alleles from FD83, FF69, GB40, and GL26, respectively, were already present in KIMDB. We also observed that more genes and alleles were predicted using KIMDB than Ramesh et al (45) database as reference (**Table 3**), probably because KIMDB has 774 sequences compared to 200 from the Ramesh et al. study.

**Table 3:** Results from mRNA MiSeq and IgDiscover.

Macaque	Origin	SHIV infection (weeks)	mRNA MiSeq library	Raw reads	Merged	Confident	Unique	Predicted reads (Ramesh et al (45) as reference)	Predicted reads (KIMDB (21) as reference)
FD83	Indian	94	IgM-only	2,356,854	708,905	708,450	158,195	68	92
FF69	Indian	42	IgM*, IgG, IgA	13,976,975	8,390,335	8,379,184	676,799	13	31
GB40	Chinese	13, 62, 79	IgM-only	2,174,703	884,603	884,172	606,763	89	107
GL26	Chinese	41	IgM-only	2,337,156	972,597	971,969	276,657	62	83

*The number of total IgM reads from FF69 was 182,910, with 37,999 unique reads.

### Comparisons of germline IGHV sequences identified by the three methods

**Figure 3:**
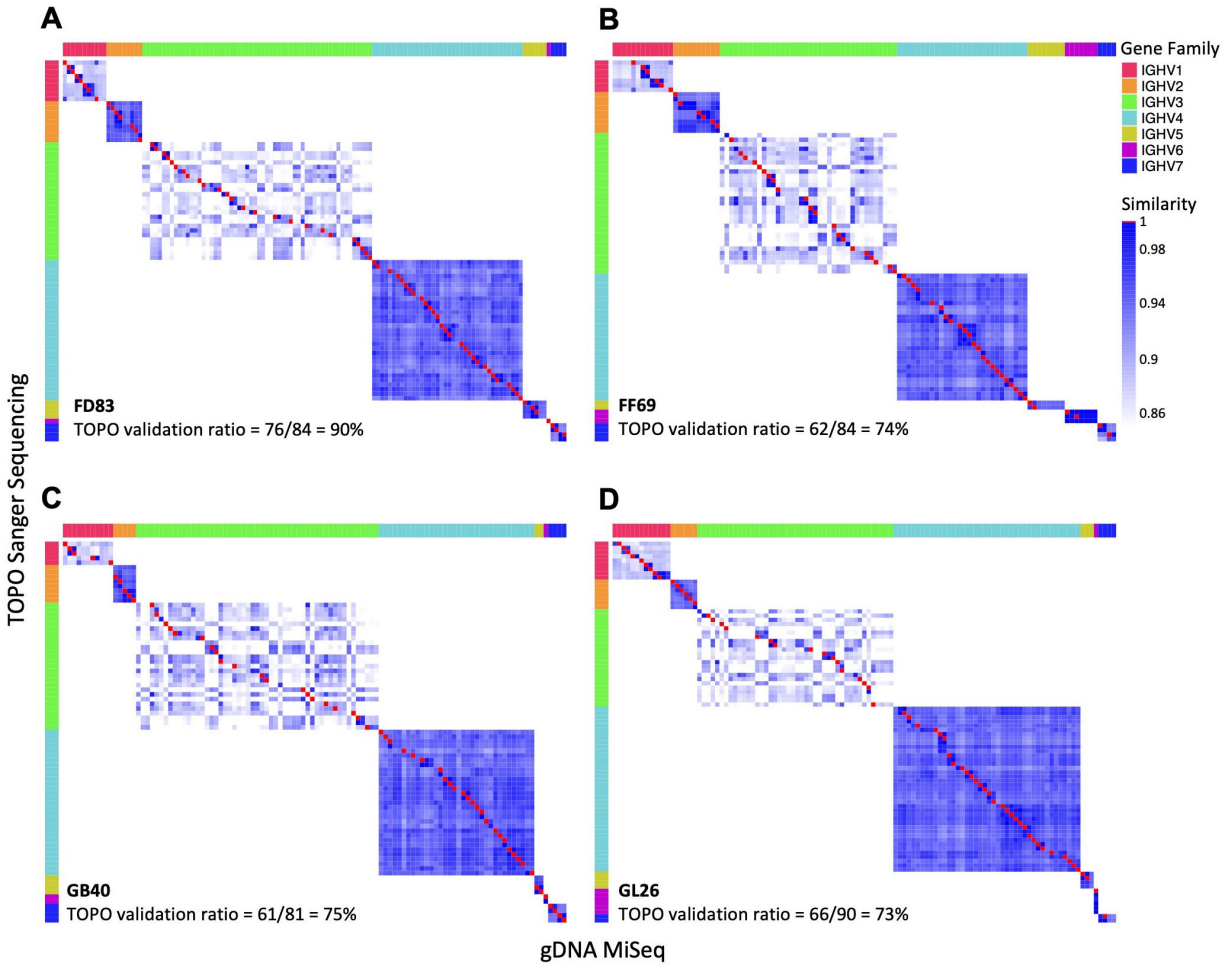
Comparison of germline genes derived from gDNA by TOPO Sanger sequencing and MiSeq. **(A–D)** Pairwise comparison of germline genes obtained from gDNA MiSeq (x-axis) and TOPO Sanger sequencing (y-axis) from each macaque. Identical allele pairs are depicted as red squares, with different gene families denoted by distinct colors. The numbers of common germline sequences between the two methods are listed in each panel.

**Figure 4:**
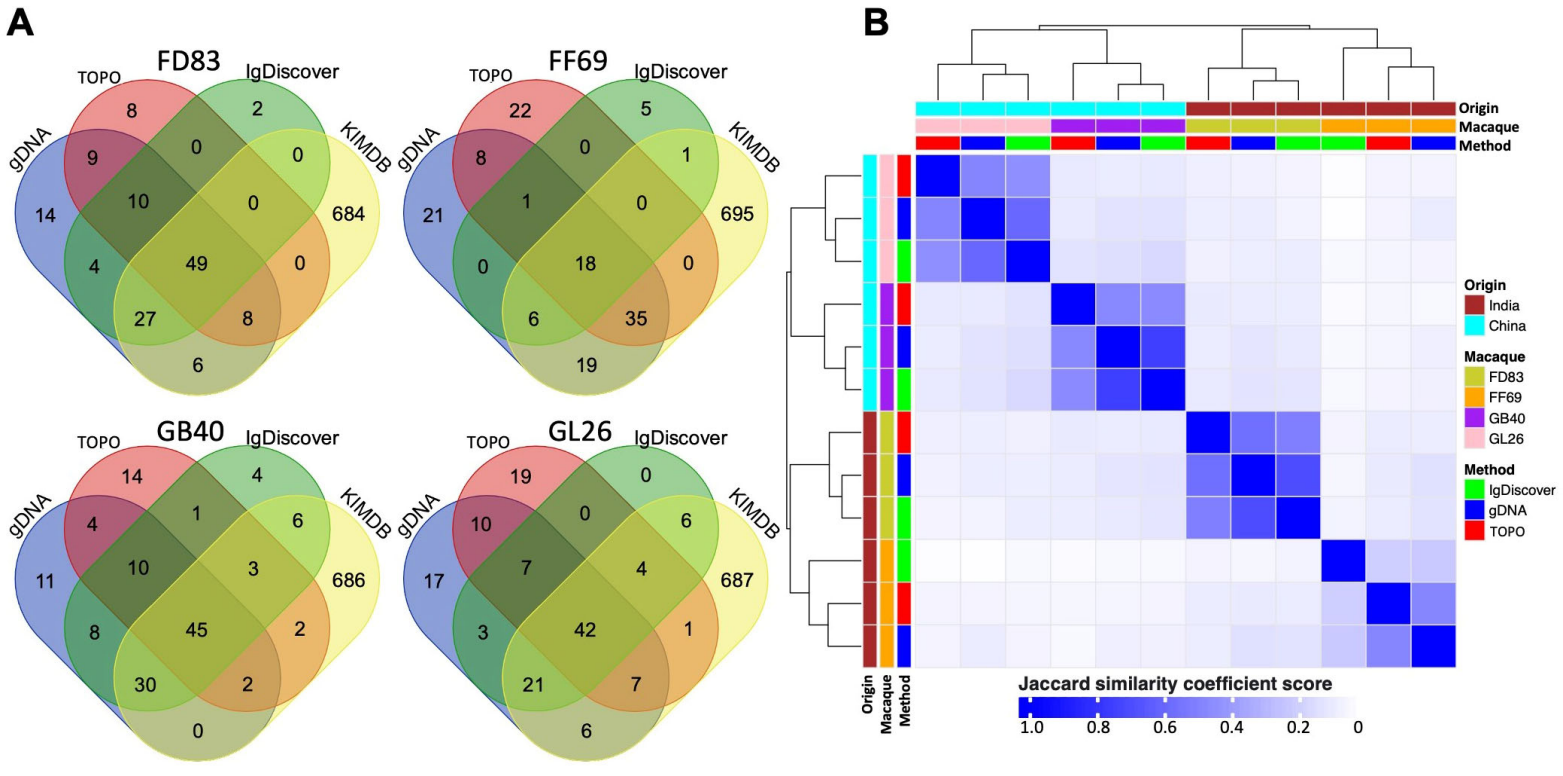
Comparative analysis of IGHV germline alleles identified through three methods and those in KIMDB. **(A)** Venn diagram illustrating the number of overlapping genes identified by TOPO sequencing (red), gDNA MiSeq (blue), IgDiscover (green), and KIMDB (yellow). **(B)** Heatmap showing the unsupervised clustering and similarity of germline alleles derived from all four macaques, as identified through gDNA TOPO sequencing, gDNA MiSeq, and IgDiscover.

Pairwise comparison of the gDNA-derived sequences revealed a high degree of concordance between TOPO and MiSeq sequencing. Specifically, N = 61 to 76 TOPO sequences were identical to those from MiSeq, resulting in validation ratios of 73% to 91% from TOPO to MiSeq (**Figure 3**). Moreover, sequences from TOPO sequencing that were not a perfect match still exhibited high similarity to MiSeq-identified sequences (>85% nucleotide identity). We calculated the sequence identity between V-genes and observed that the IGHV1 and IGHV3 family genes have the lowest intra-family identity (~90% or below) (**Figure 3**). A total of N = 90 (98%), 25 (81%), 97 (91%), and 77 (93%) alleles from IgDiscover were corroborated by either gDNA TOPO or MiSeq in each respective macaque (**Figure 4A**). In each macaque, 11 or more IGHV alleles identified via gDNA TOPO or MiSeq were missing in IgDiscover output and KIMDB. Moreover, several sequences predicted by IgDiscover were not observed in the results from gDNA TOPO, MiSeq, or KIMDB. To further evaluate IgDiscover’s predictive power, we compared the sequences from IgDiscover with those from gDNA MiSeq and TOPO sequencing using the Jaccard similarity coefficient (**Figure 4B**). The alleles from IgDiscover clustered consistently with the other two methods for each macaque, validating IgDiscover’s predictive power. Notably, while the two Indian-origin and two Chinese-origin macaques clustered separately, intra-origin similarities were not dramatic, underscoring the germline gene diversity among individual macaques. To examine whether the reference database plays a role in IgDiscover’s performance, we conducted an analysis altering only the input reference for IgDiscover. Instead of KIMDB, we utilized the 200 germline alleles from Ramesh et al (45). The intra-origin similarities were largely consistent, with the primary difference being the number of predicted alleles by IgDiscover between the two runs (**Table 3**; **Supplementary Figure S2**).

### Germline IGHV database updated for rhesus macaques

**Figure 5:**
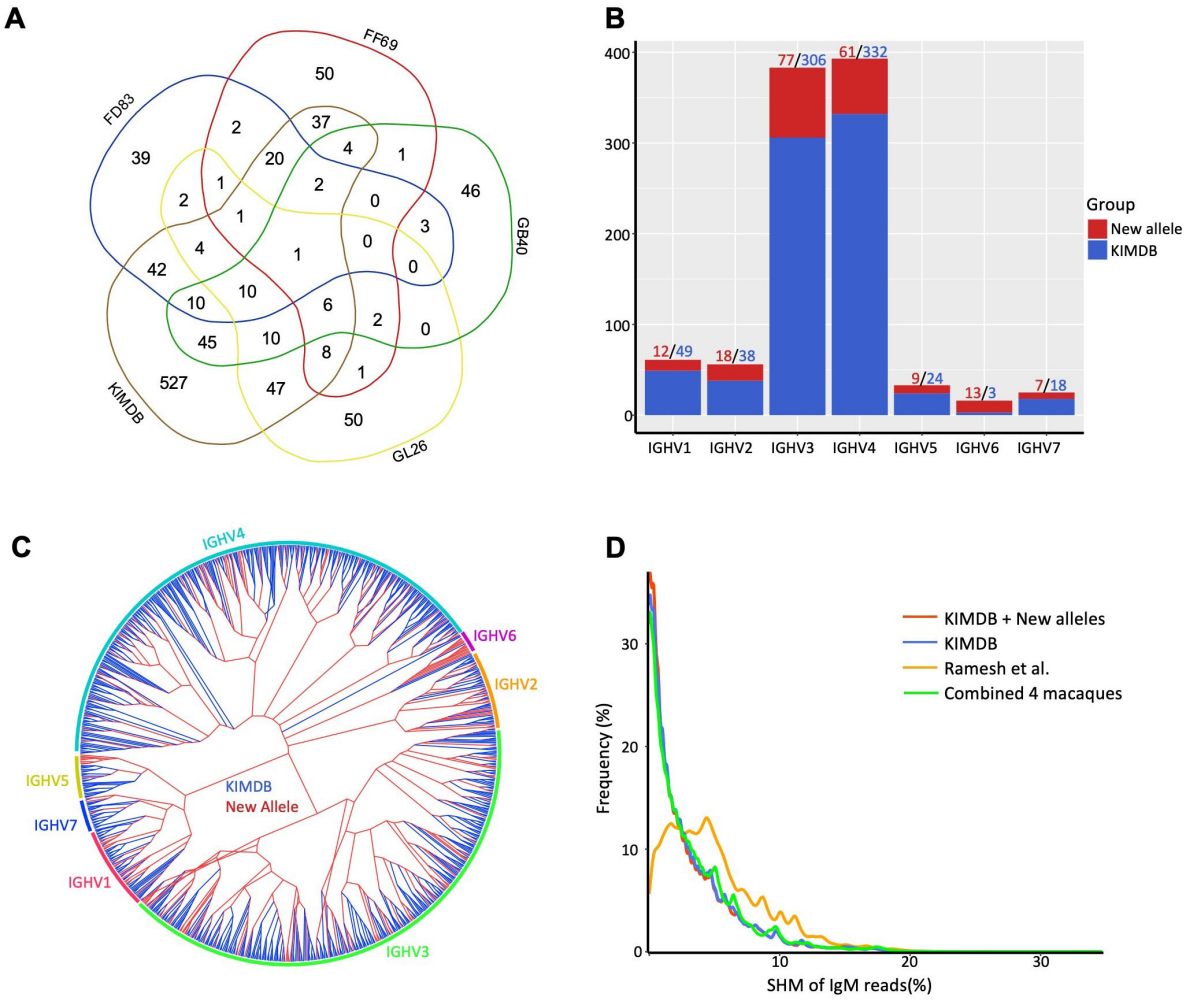
Cross-donor prevalence and gene family distribution of rhesus IGHV genes. **(A)** Venn diagram of germline genes shared between this study and KIMDB. For each macaque, the germline genes identified by all three methods are combined. **(B)** Number of new rhesus IGHV alleles identified for all gene families. **(C)** Phylogenetic tree of the extended rhesus germline database, with the alleles in KIMDB colored in blue and the new alleles in red. **(D)** The updated rhesus germline database reduces the SHM levels of IgM repertoire when compared to previous databases.

We consolidated alleles identified from gDNA TOPO sequencing, gDNA MiSeq, and IgDiscover to construct individual germline alleles for each macaque. This resulted in final allele counts of N = 137, 136, 140, and 143 for FD83, FF69, GB40, and GL26, respectively. These allele sets were then compared to those in KIMDB (**Figure 5A**). Remarkably, only the IGHV6-1*02 allele was common across all four macaques and KIMDB. FD83, FF69, GB40, and GL26 shared N = 47 (34%), 37 (27%), 45 (32%), and 42 (30%) alleles with KIMDB, respectively. Conversely, N = 39 (29%), 50 (37%), 46 (33%), and 50 (35%) alleles were unique to each respective macaque. Additionally, N = 527 (68%) alleles in KIMDB were not observed in any of the macaques, and N = 197 germline alleles identified from these four macaques were not present in KIMDB, IMGT, or Ramesh et al. databases. Of the 197 newly identified, 116 (59%) were validated by at least two methods, and 143 (73%) were found in at least two macaques or two independent sample sources (**Supplementary Table S1**). The overall validation rates were above 90%, with the highest for IgDiscover – 100% in FD83 and GL26, 98% in GB40, and 87% in FF69, followed by gDNA TOPO – 98% in GB40 and GL26, and 92-94% in FF69 and FD83, and the last by gDNA MiSeq – 96-97% in GL26 and GB40, 92% in FD83, and 86% in FF69. The 197 new alleles spanned all seven IGHV gene families, with IGHV3 and IGHV4 families having the highest numbers of new allele counts. Notably, we discerned N = 13 additional alleles from the IGHV6-1 gene, in contrast to the N = 3 IGHV6-1 alleles in KIMDB (**Figure 5B**). These newly identified IGHV6-1 alleles were more like IGHV6-1*02, exhibiting a T72S (ACC to AGC) substitution relative to IGHV6-1*01 (**Supplementary Figure S3**). A dendrogram was generated to show the relationships between these new alleles and KIMDB, revealing a relatively uniform distribution of new alleles across gene families, except for the IGHV6 cluster (**Figure 5C**). We then integrated the newly identified germline alleles with KIMDB to establish an enhanced germline database for rhesus macaques. To evaluate the improvements of this updated database, we analyzed the SHM levels of the IgM transcripts, comparing results against the updated database, KIMDB, and the database from Ramesh et al (45). The updated database consistently presented the lowest SHM levels in IgM reads, with the majority nearing zero. This performance was marginally superior to KIMDB but markedly improved over the germline database from Ramesh et al (45) (**Figure 5D**). Subsequently, we re-annotated the sequence reads derived from gDNA MiSeq to these three germline databases to examine allelic distances and MiSeq sequencing errors. The levels of nucleotide mismatches mirrored the trends observed in the IgM transcripts, with the updated databases again displaying the lowest mismatch levels (**Supplementary Figure S4A**).

### Individualized macaque germline alleles

**Figure 6:**
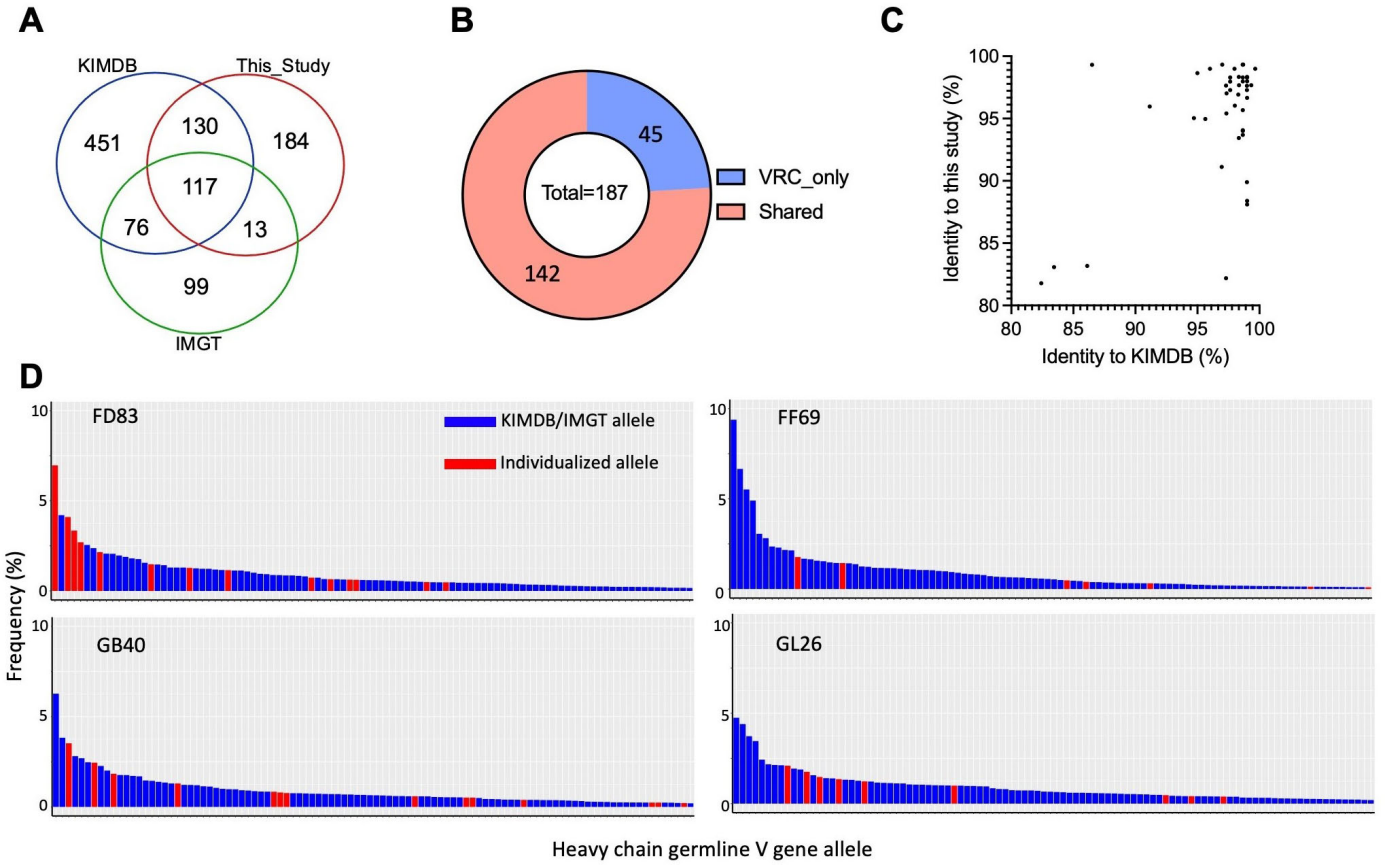
Divergence and usage frequency of rhesus germline genes. **(A)** Venn diagram of all germline alleles between this study, IMGT, and KIMDB. **(B)** 142 IGHV alleles from the VRC study are found in IMGT and KIMDB. 45 alleles are unique to the VRC study. **(C)** Sequence identity of IGHV alleles from the VRC study in comparison to those identified in this study and those in KIMDB. **(D)** The majority of frequently used germline genes in each macaque are included in KIMDB. However, multiple individualized alleles are also frequently used in the expressed naïve BCR repertoires. The x-axis displays the top 100 most frequently used germline alleles in each macaque; the y-axis represents the frequency of germline usage in the naïve BCR repertoire of each macaque.

To better understand the range and enhancements of the updated database in capturing the extensive IGHV allelic diversity observed in rhesus macaques, we compared the germline alleles identified from the four rhesus macaques in this study to those in the KIMDB database and IMGT’s functional alleles (**Figure 6A**). Our analysis revealed that N = 117 germline alleles were consistent across all three databases. However, there were notable differences in unique allele counts: N = 451 (58%) were exclusive to KIMDB, N = 184 (41%) were found only in this study, and N = 99 (48%) were unique to IMGT. This significant proportion of unique alleles underscores the allelic diversity of IGHV in rhesus macaques. To further examine the coverage of germline alleles by these databases, N = 187 germline alleles were predicted by IgDiscover using the IgM transcripts from another four Indian rhesus macaques in a previous study from the Vaccine Research Center (VRC) (42). The comparison showed that N = 142 alleles were found in the existing databases, whereas N = 45 (24%) were unique to the VRC study (**Figure 6B**; **Supplementary Figure S4B**). We then analyzed the nucleotide sequence identity of the VRC macaque-specific alleles against the closest matches in this study and KIMDB. Most alleles were highly similar yet not identical to those in either this study or KIMDB, reflecting the diverse germline alleles in rhesus macaques. Notably, three alleles (7%) exhibited less than 85% identity with any alleles in KIMDB, IMGT, and this study, suggesting previously unidentified germline genes or alleles upon validation (**Figure 6C**). Additionally, we investigated the usage frequencies of macaque-specific alleles identified in this study and compared them with alleles shared with KIMDB (**Figure 6D**). We observed that some macaque-specific germline alleles exhibited high expression levels across all analyzed macaques. For instance, the FD83-specific germline IGHV4-NL_33*01_W8423 is one of the most frequently (7%) used IGHV alleles in FD83, highlighting the importance of sampling individual macaques for their unique IG germline sequences for downstream matured antibody repertoire analyses.

## Discussion

The immune system of rhesus macaques has long been a focal point of scientific inquiry primarily due to its close phylogenetic relationship to that of humans and its pivotal role in biomedical research (42, 47). As the optimal animal model for HIV-1 infection, rhesus macaques have been infected with SHIV for studies of broadly neutralizing antibody development (32, 33). Due to high polymorphism and copy number variation of antibody gene locus in macaques, each macaque may contain about 100 to 140 IGHV germline alleles (45), with a subset of them potentially being unique to each animal. To interrogate antibody SHM and affinity maturation in macaques, a comprehensive determination of the rhesus IGHV germline sequences is required.

From the four rhesus macaques of interest, we applied the gDNA TOPO sequencing, gDNA MiSeq, and mRNA MiSeq with IgDiscover to determine the IGHV germline genes from each animal and illustrate the detection power of each method. The variations of germline genes captured through the three methods emphasize the intricate polymorphism of the BCR genetics of rhesus macaques. The high validation ratios of over 70% for identical sequences obtained from gDNA TOPO sequencing and gDNA MiSeq reinforce the reliability of the employed methodologies. In this study, gDNA MiSeq identifies the most germline genes, which may be due to the high sequencing depth. However, the presence of sequencing errors may leave the accuracy of sequences uncertain. To address this uncertainty, we identified the necessary sequencing depth cutoffs to exclude low-quality and less confident sequences. In the future, the incorporation of a unique molecule identifier (UMI) to gDNA MiSeq could further reduce sequencing errors and improve the quality and accuracy of MiSeq-identified germline genes. TOPO Sanger sequencing of gDNA has a high and reliable sequencing quality but is labor intensive and low throughput, resulting in up to 80% coverage of those determined by gDNA MiSeq. Ideally, the Miseq-derived alleles should be explicitly validated by Sanger sequencing. Otherwise, those not validated can only be regarded as an approximation to the actual alleles by the imposition of a sequencing depth threshold for high confidence. Hence, further work would be required to establish MiSeq as a reliable gDNA-based method for identification of IGHV alleles.

Unlike gDNA sequencing where non-rearranged alleles can be included, IgDiscover provides direct evidence of allele expression and usage. Another key advantage of mRNA-based inference is 5’RACE, which minimizes the potential bias in PCR primer design. Although capturing on average about 70% of germline sequences from gDNA MiSeq, IgDiscover has been effective in IG germline inference and less sensitive to sequencing errors benefiting from the algorithm’s statistics that discriminate gene polymorphisms from SHM. The limitations of IgDiscover lie in the number of expressed transcripts of naïve BCRs and V-gene usage (48), especially those with low levels of usage. For example, the 13 novel IGHV6-1*02 alleles that IgDiscover did not predict were expressed at a low level of 0.01% in the mRNA MiSeq data in these macaques. Additionally, FF69 had only 31 germline alleles predicted by IgDiscover due to the limited number of naïve BCRs sequenced. Hence, sequencing more naïve B cells and IgM-only libraries would improve the number of predicted germlines. Finally, the performance of IgDiscover is affected by the reference sequences applied, and a more comprehensive reference database would enable greater prediction power from IgDiscover. Both gDNA TOPO sequencing and gDNA MiSeq are robust to these limitations because they sequence the gene-targeted PCR amplicons from the genomic loci of non-B cells instead of the expressed BCRs on naïve B cells.

Our research offers a significant contribution to the existing germline gene resources, complementing established rhesus macaque germline databases like KIMDB and IMGT. Our updated database exhibits enhanced performance compared to KIMDB alone. It also underscores potential areas for further enhancement in current germline databases. We posit that KIMDB remains a database suitable for most studies, especially those not requiring precise germline allele assignment. However, comparing alleles from the VRC study (42) with those in current IGHV databases continues to show that each rhesus macaque contains about 20% germline alleles not covered by KIMDB, with each allele showing up to 5% diversity (excluding the most distant three alleles) to the closest homolog alleles in KIMDB. Multiple individual macaque-specific alleles show high frequencies of usage in antibody repertoire. All these results highlight the need to identify individualized macaque germline alleles for studies that require precise germline assignments, e.g., germline BCR activation (49, 50), pathways of antibody lineage development (51), and functions of SHM (52). Sequencing germline genes from more rhesus macaques is still needed to provide more comprehensive coverage of rhesus IGHV germline gene diversity. Although the IGHV gene polymorphism is lower in humans than in rhesus macaques, diversity still exists, e.g., in African populations, that should not be minimized, especially in HIV-1 broadly neutralizing antibody studies.

The observed differences between the two Indian (FD83 and FF69) and the two Chinese (GB40 and GL26) rhesus macaques are not more pronounced than those observed among individual macaques. This result suggests that geographical variation might not be the primary determinant of B cell germline alleles in rhesus macaques. However, our study is limited to only four rhesus macaques analyzed and to the PCR amplicons and mRNAs sequenced. The PCR primers used may not cover all IGHV alleles. The need for a broader validation of the database by more rhesus macaques is being addressed by large scale studies involving the IG gene sequencing of more than a hundred NHPs from multiple AIDS-designated breeding colonies in the US (presented at the 41st Annual Symposium on NHP Models for AIDS). While our datasets provide a valuable resource to the database, we recognize that the rhesus IGHV germline genes are likely still unsaturated. Without locations in the genome, the newly identified germline sequences cannot be precisely determined as new genes or alleles.

In conclusion, our findings reinforce the crucial role of rhesus macaques in advancing our understanding of B cell immunology in human diseases. The insights gained in this study would directly impact the antibody lineage analyses with SHM in these four macaques. Likewise, the addition of validated germline sequences would facilitate a more in-depth and more informed comparative study using rhesus macaque models, potentially influencing vaccine development, antibody therapeutics, and immune genetics. As sequencing technologies evolve, we anticipate a more refined and expansive understanding of the immune repertoires in rhesus macaques and other species with close phylogenetic ties to humans, such as swine.

## Data Availability

The datasets presented in this study can be found in online repositories at GenBank under accession numbers PQ429293 to PQ430403, at the figshare link: https://figshare.com/articles/dataset/Combined_Rhesus_Macaque_germline_database/27170985?file=49615155, and the NCBI SRA database under accession number PRJNA1181892.
