# Supplementary material for: Novel rhesus macaque immunoglobulin germline genes identified by three sequencing approaches
Source: Front Immunol. 2024 Dec 24;15:1506348. doi: 10.3389/fimmu.2024.1506348 (PMC11703713; doi:10.3389/fimmu.2024.1506348)
Supplement: Supplementary file 1 [file Image1.pdf]

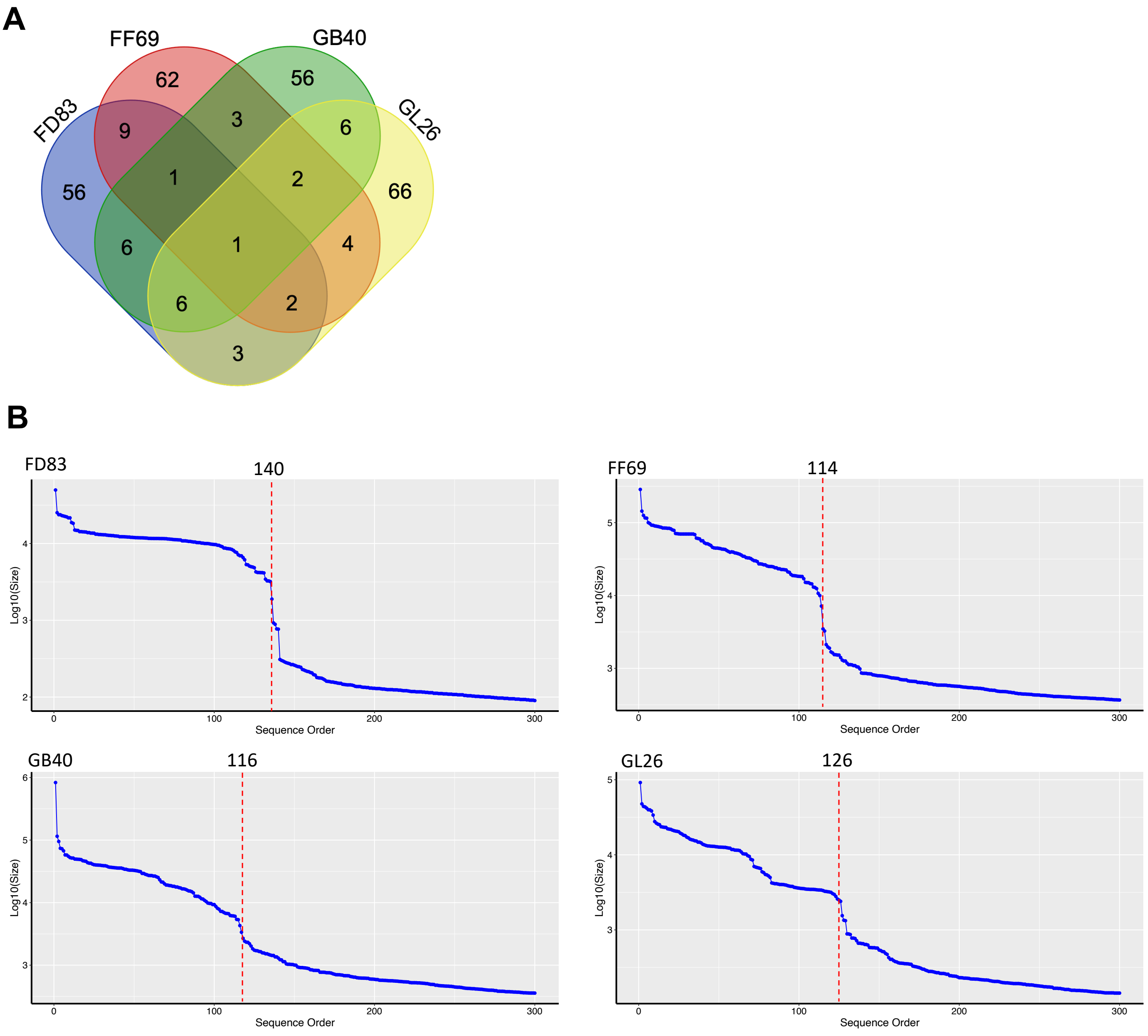

Figure S1. Germline alleles identified via gDNA TOPO and MiSeq sequencing. A The Venn diagram illustrates the overlap of germline alleles identified by TOPO sequencing in four macaques. **B** Distribution of unique germline gene sequencing depth from gDNA sequencing for each macaque. The sequences were ordered by sequencing depth (number of reads). The red dashed lines indicate the cutoff for number of sequences selected for further validation.

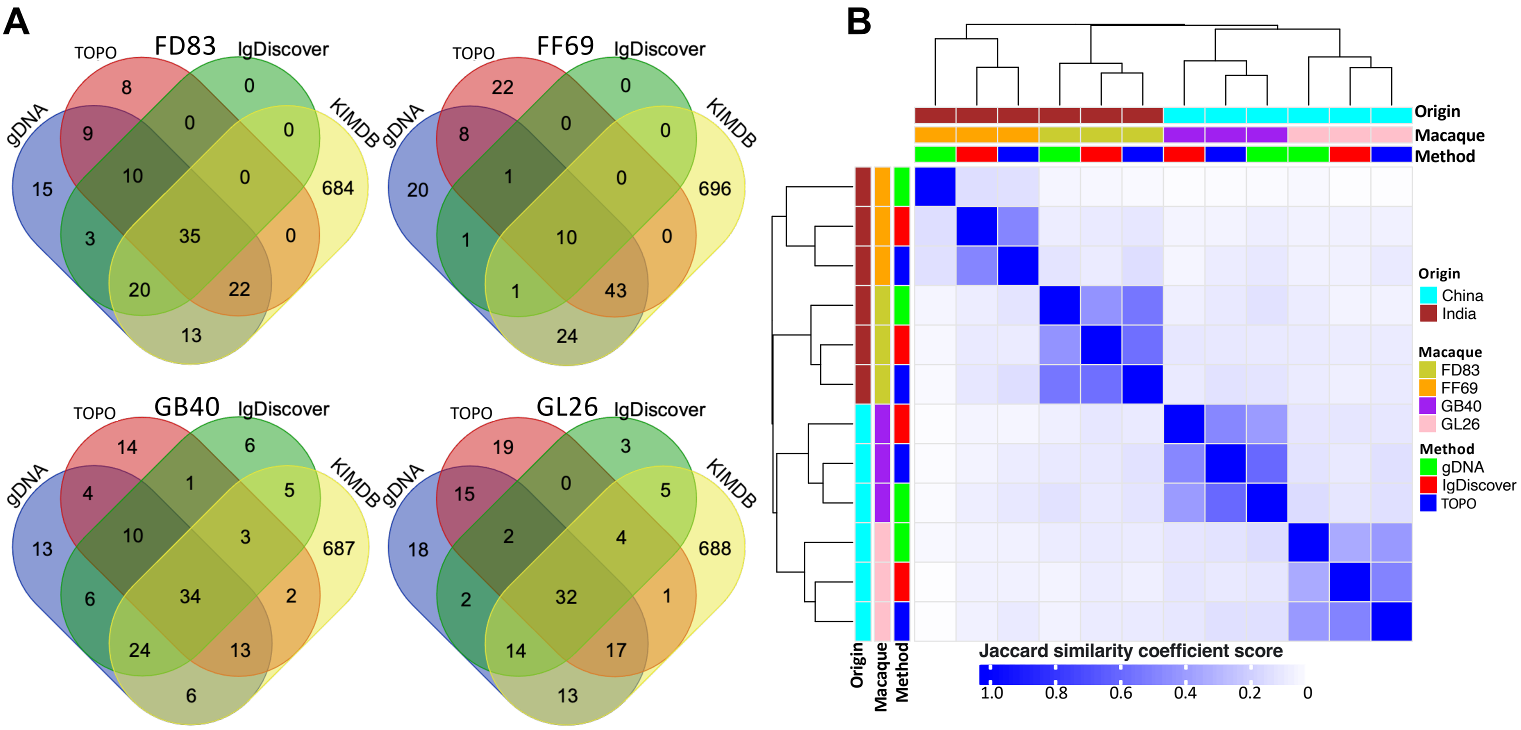

Figure S2. Comparative analysis with an alternative IgDiscover result using Ramesh et al. as the initial reference. **A** The Venn diagram illustrates the number of overlapping genes identified by TOPO sequencing (red), gDNA MiSeq (blue), IgDiscover using Ramesh et al as reference (green), and KIMDB (yellow). **B** Heatmap shows the unsupervised clustering and similarity of germline alleles derived from all four macaques, as identified through gDNA TOPO sequencing, gDNA MiSeq, and IgDiscover using Ramesh et al. as reference.

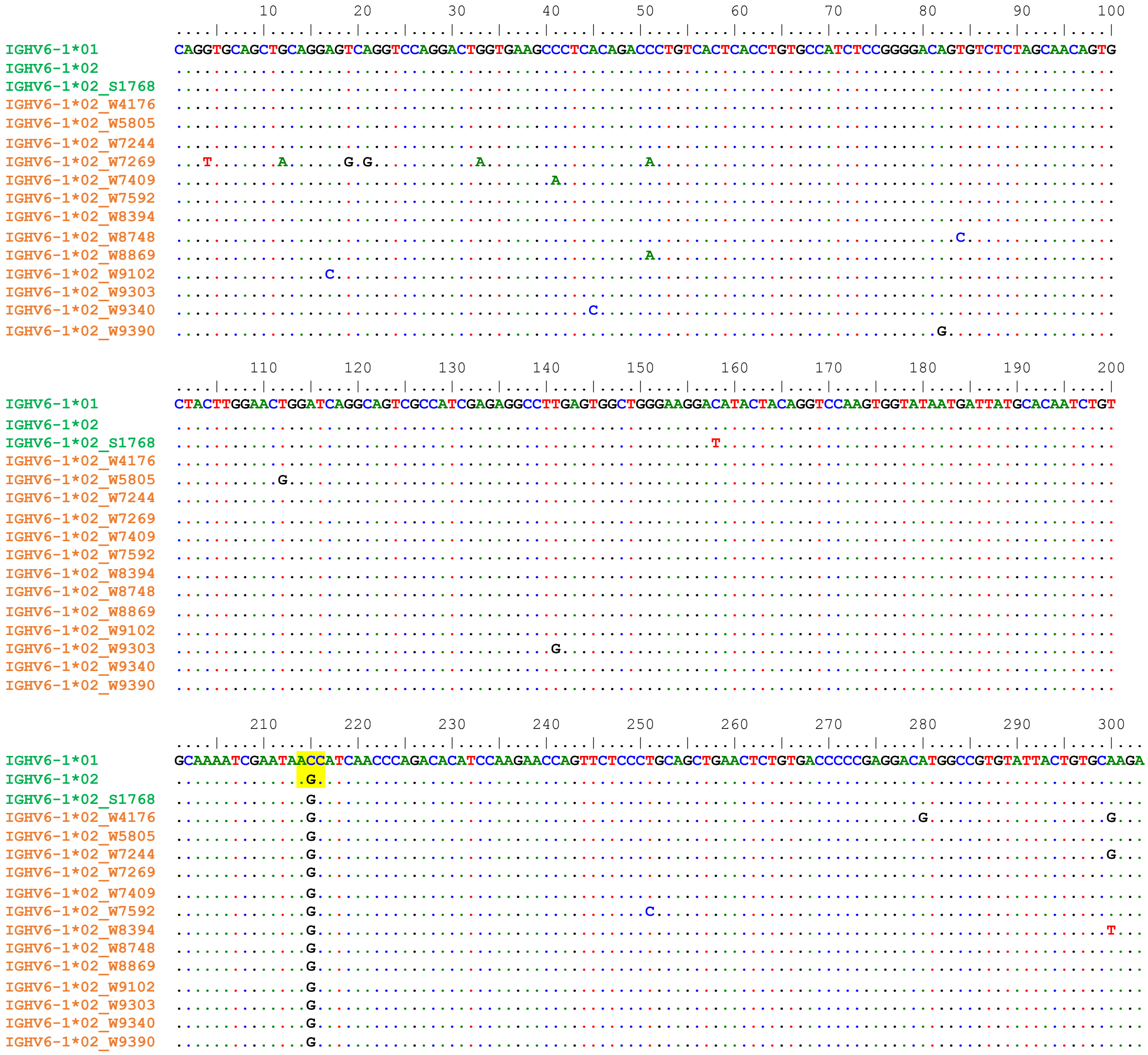

Figure S3. Allelic polymorphism of the rhesus IGHV6-1 gene. Nucleotide sequence alignment of IGHV6-1 alleles in KIMDB (green) and new alleles (orange) identified in this study. The codons for a characteristic T72S substitution between IGHV6-1*01 and IGHV6-1*02 are highlighted in yellow.

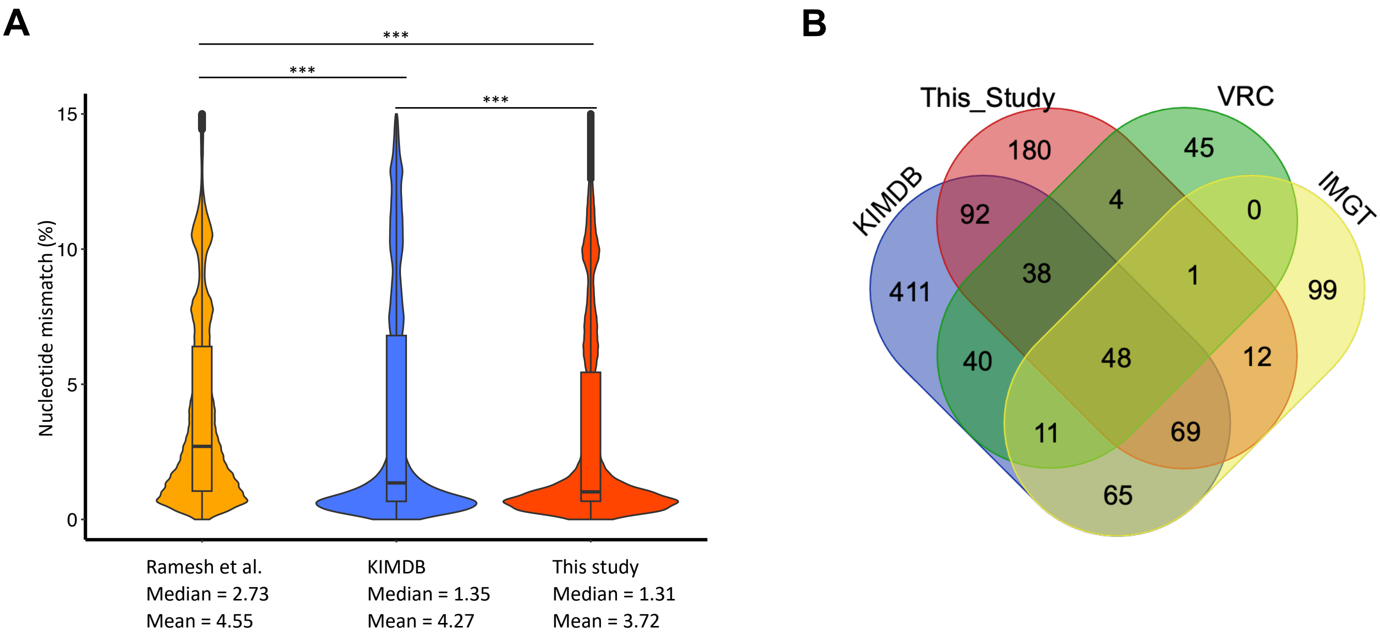

Figure S4. Comparisons of updated IGHV germline databases. A The updated rhesus IGHV germline database reduces the levels of nucleotide mismatches in gDNA MiSeq-derived reads. ***: Wilcoxon rank-sum test, p< 0.001. **B** Shared alleles in KIMDB, VRC study, this study, and IMGT.
